# Supplementary material for: FGF4-FGFR1 signaling promotes podocyte survival and glomerular function to ameliorate diabetic kidney disease in male mice
Source: Nat Commun. 2025 Nov 25;16:10430. doi: 10.1038/s41467-025-65978-4 (PMC12647821; doi:10.1038/s41467-025-65978-4)
Supplement: Supplementary file 1 — Supplementary Information [file 41467_2025_65978_MOESM1_ESM.pdf]

Supplementary Information

The FGF4-FGFR1 signaling promotes podocyte survival and glomerular function to ameliorate diabetic kidney disease

Jie Zhou[#*], Shuxin Wang[#], Jiaxin Lou[#], Beibin Pan, Min Zhao, Qian Li, Jing Zhou, Yali Du, Shuodan Ding, Meiling Yu, Jingjing Zhou, Xinwei Chen, Lingwei Jin, Xinyi Wang, Yepeng Hu, Zhe Wang, Xiaokun Li, Chao Zheng[*], Jian Sun[*], Zhifeng Huang[*]

[#]These authors contributed equally to this work

*Correspondence author: hzf@wmu.edu.cn (Z.H.), sunjian@wmu.edu.cn (J.S.), zhoujie123@wmu.edu.cn (J.Z.), and chao_zheng@zju.edu.cn (C.Z.).

This PDF file includes:

Supplementary Figures 1 to 13

Supplementary Tables 1 to 2

Supplementary Fig. 1

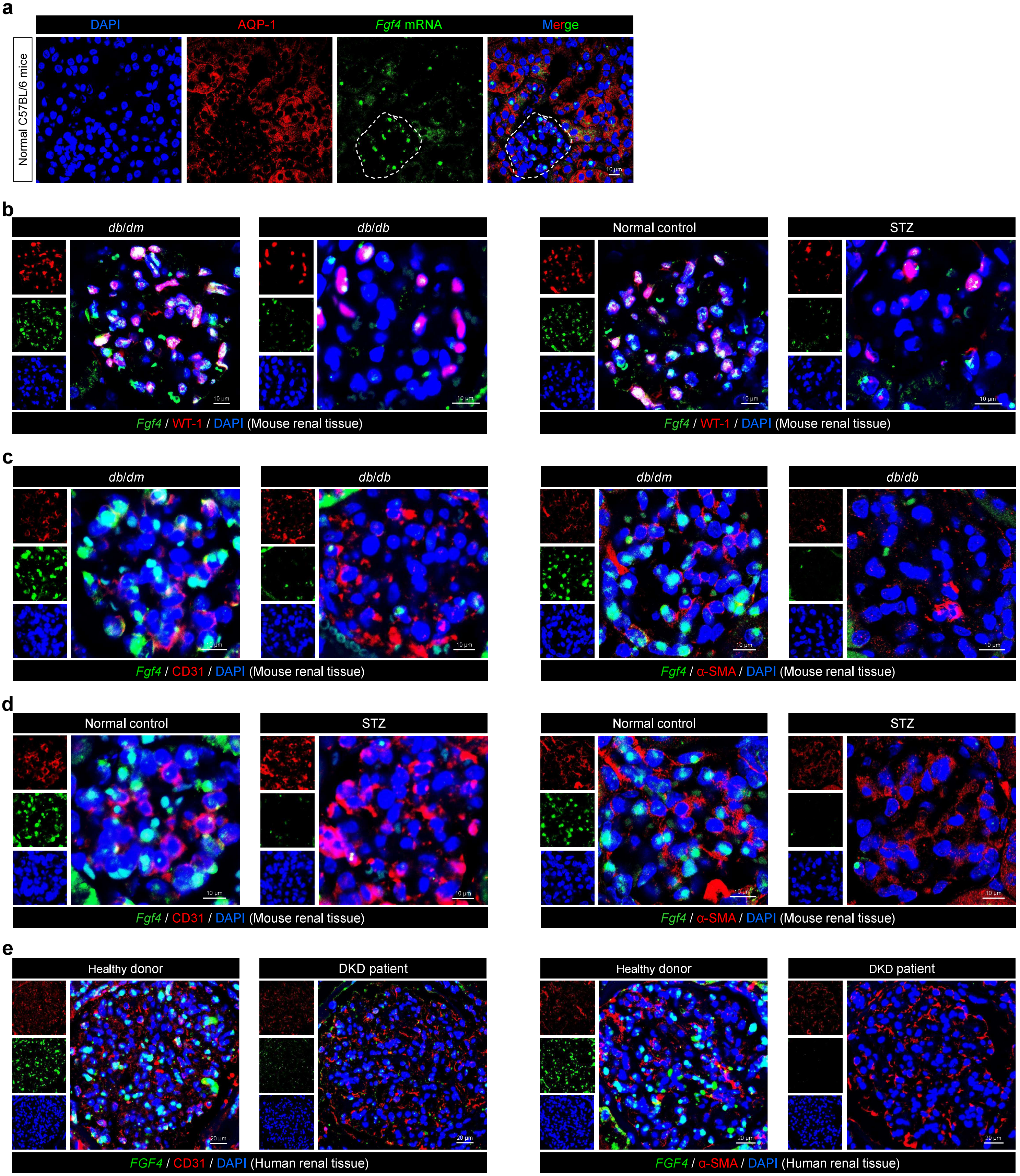

Supplementary Fig. 1. Expression pattern of FGF4 in DKD models and human renal tissues.

a, Dual immunofluorescence demonstrating co-localization of *Fgf4* mRNA (green) with the tubular marker AQP-1 (red) in renal tissues from C57BL/6 mice. with white dashed lines demarcating glomerular boundaries. **b**, Co-localization analysis of *Fgf4* mRNA (green) with the podocyte marker WT-1 (red) in DKD models. **c**,**d**, Co-localization of *Fgf4* mRNA (green) with the mesangial cell marker α-SMA (red) or endothelial cell marker CD31 (red) in kidneys from (**c**) *db*/*db* and (**d**) STZ-induced diabetic mice. **e**, Co-localization of *Fgf4* mRNA (green) with α-SMA (red) or CD31 (red) in renal tissues from healthy donors and DKD patients. Nuclei were counterstained with DAPI (blue). DKD, diabetic kidney disease; AQP-1, aquaporin 1; α-SMA, alpha smooth muscle actin; WT-1, Wilms' Tumor 1.

Supplementary Fig. 2

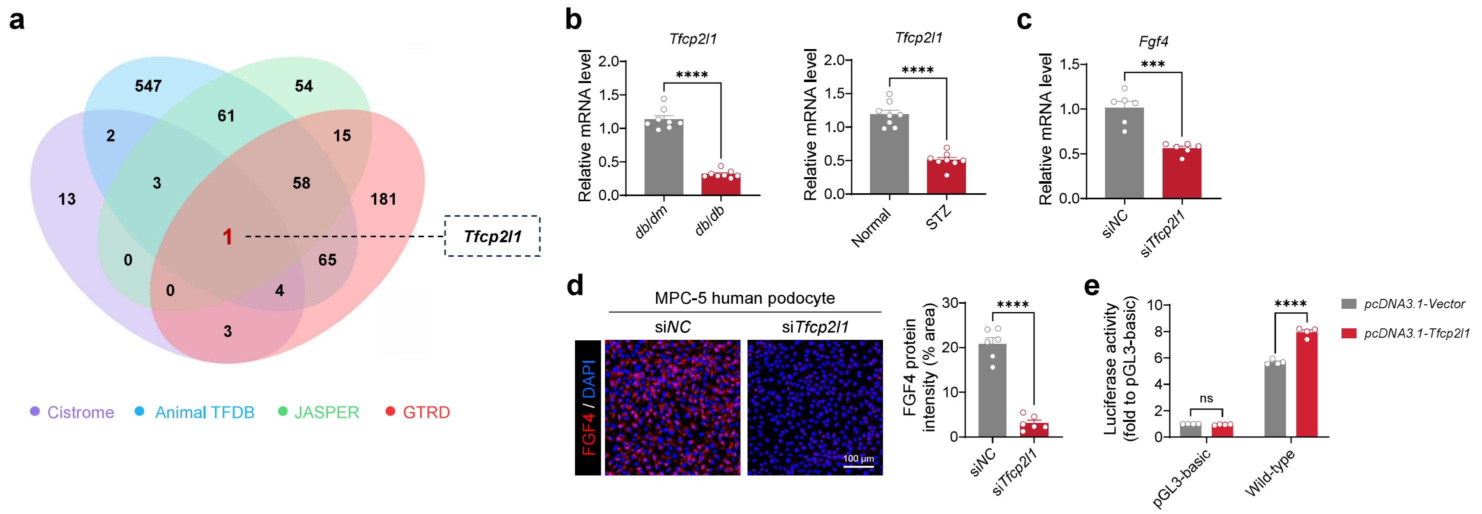

Supplementary Fig. 2. Regulation of *Fgf4* mRNA expression by the transcription factor TFCP2L1. a, Prediction of transcription factors potentially regulating FGF4 expression in the kidney. **b**, Relative mRNA levels of the transcription factor *Tfcp2l1* in the kidneys of *db/db* and STZ-induced diabetic mice. **c**, *Fgf4* mRNA level in MPC-5 cells transfected with si-*NC* or si*Tfcp2l1* (n = 6). **d**, Representative image and quantification of MPC-5 cells stained with FGF4 antibody after transfection with si*NC* or si*Tfcp2l1* (n = 6). **e**, Luciferase reporter activity of *Fgf4* promote in HEK-293T cells transfected with *Tfcp2l1* plasmid (n = 4). Data are presented as mean ± s.e.m. $^{***}p < 0.001$, $^{****}p < 0.0001$ as determined by unpaired two-tailed Student's *t*-test (**b-d**) or ordinary two-way ANOVA followed by Sidak's multiple comparisons tests (**e**). NC, negative control.

Supplementary Fig. 3

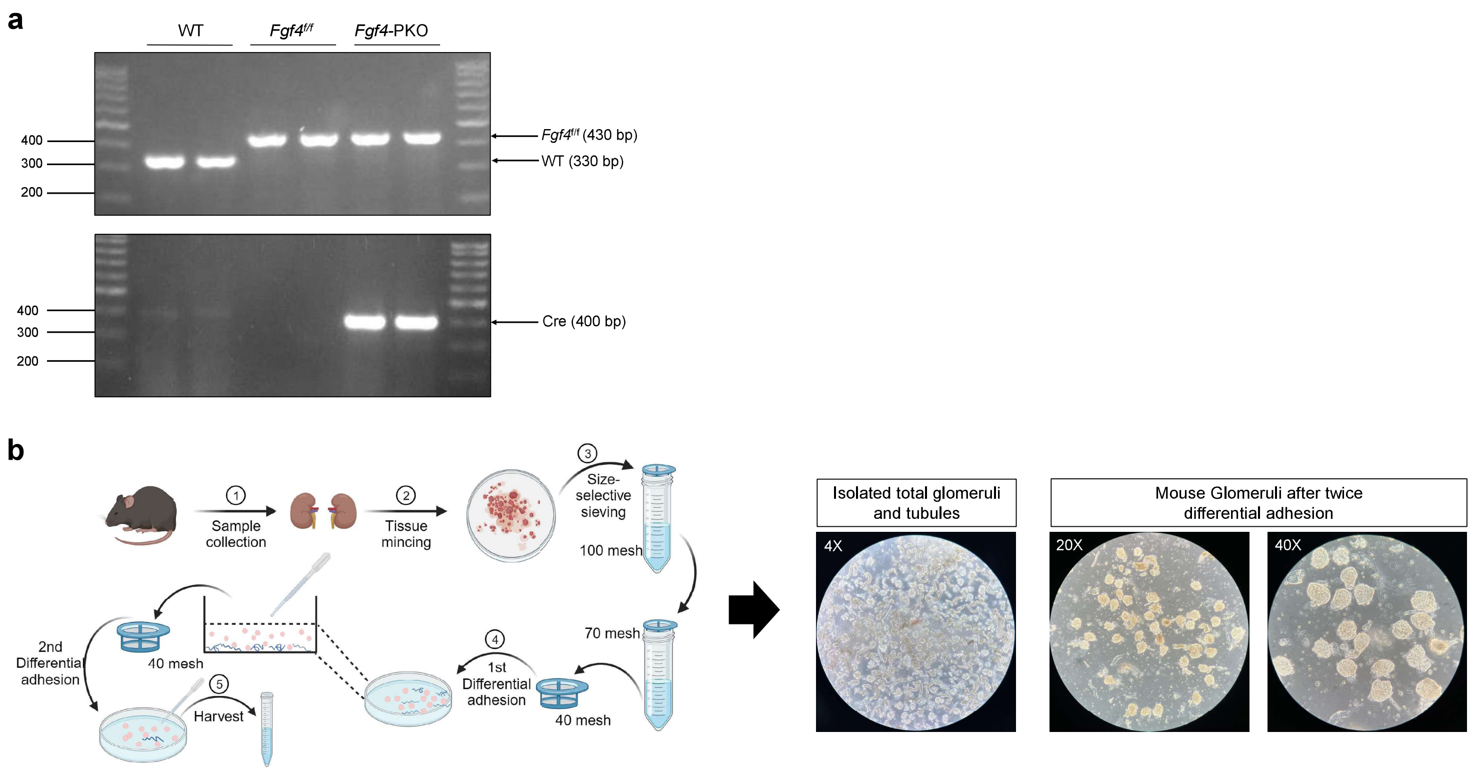

Supplementary Fig. 3. Validation of podocyte-specific *Fgf4* knockout mice and glomerular isolation. a, PCR genotyping confirms successful generation of podocyte-specific *Fgf4* knockout mice. Representative gel electrophoresis images show WT, floxed (fl/fl), and KO genotypes for target gene. **b**, Schematic representation of glomerular tissues isolated from different experimental groups using selective sieving. bp, base pair; WT, wild-type; KO, knockout.

Supplementary Fig. 4

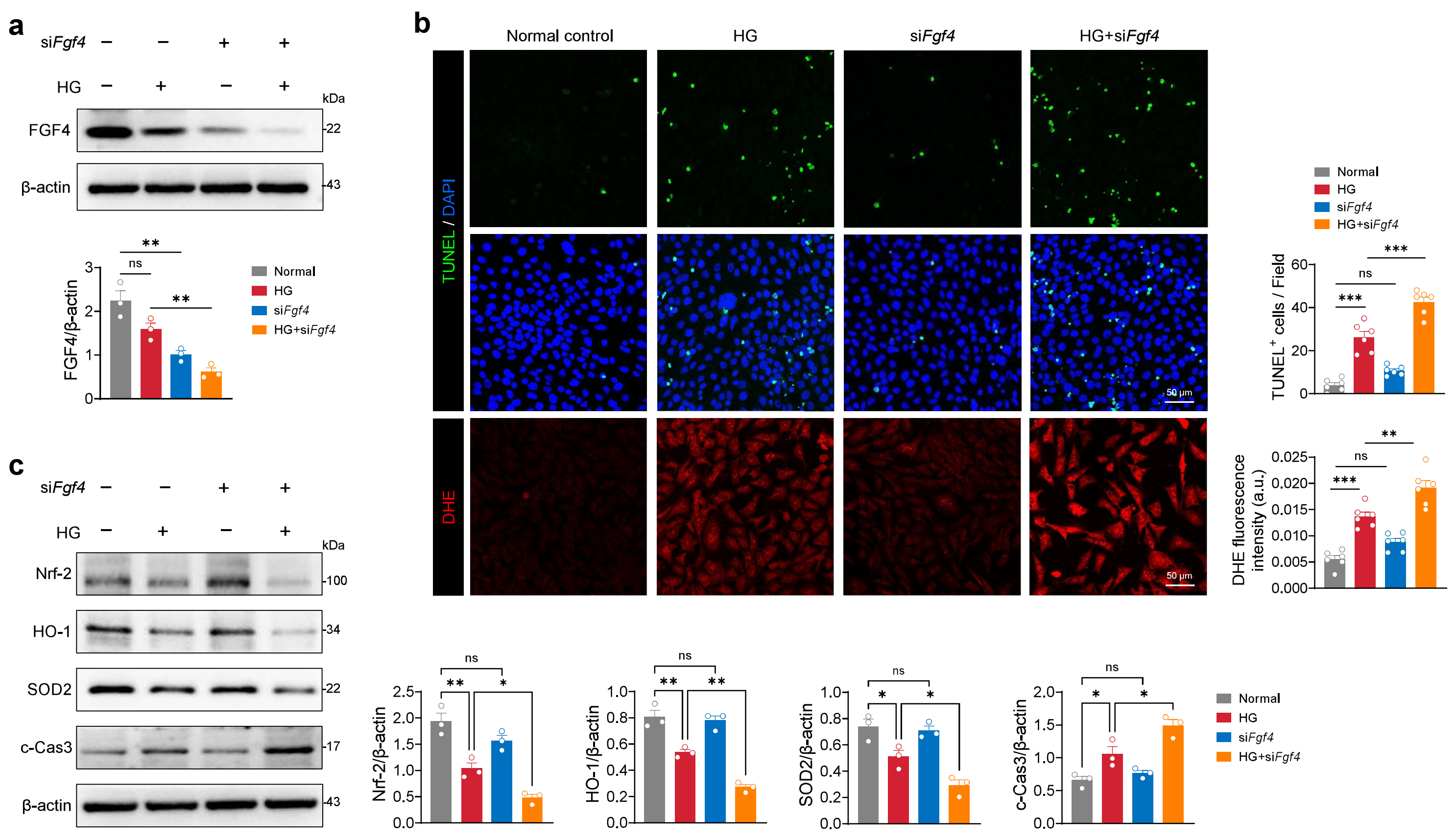

Supplementary Fig. 4. Impact of FGF4 knockout in MPC-5 cells on high glucose-induced apoptosis and oxidative stress. a-c, MPC-5 cells were transfected with *Fgf4* siRNA or control siRNA for 24 hours, followed by incubation with 33.3 mM high glucose for an additional 24 hours. **a**, Western blot analysis and quantification of FGF4 expression in total MPC-5 cell lysates (n = 3). **b**, Representative images and quantification of MPC-5 cells stained with TUNEL (green) or DHE (red) in the indicated experimental groups (n = 6). Nuclei were counterstained with DAPI (blue). **c**, Western blot analysis and quantification of proteins related to antioxidant activity and apoptosis in total MPC-5 cell lysates (n = 3). β-actin was used as a loading control. Data are presented as mean ± s.e.m. $^{*}p < 0.05$, $^{**}p < 0.01$, $^{***}p < 0.001$ as determined by ordinary one-way ANOVA followed by Tukey's multiple comparisons tests; ns, not significant.

Supplementary Fig. 5

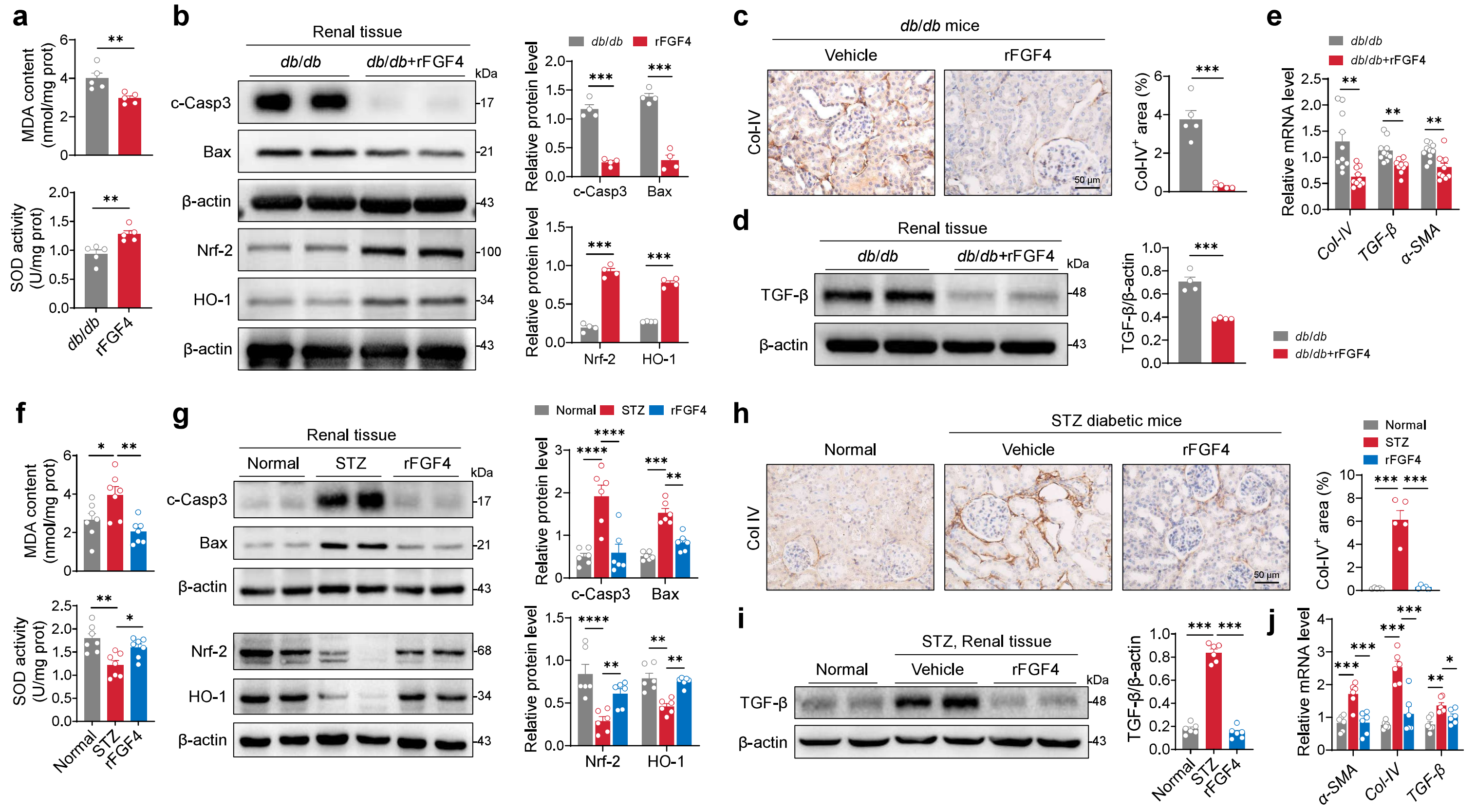

Supplementary Fig. 5. rFGF4 alleviates renal oxidative stress, apoptosis, and fibrosis in diabetic mice. rFGF4 attenuates diabetic renal injury in (**a-e**) *db/db* and (**f-j**) STZ-induced diabetic mice. **a**, Renal MDA and SOD levels (*n* = 5). **b**, Immunoblotting and quantification of c-Cas3, Bax, Nrf-2, and HO-1 (*n* = 4). **c**, Immunohistochemical staining of Col-IV. **d**, Immunoblotting and quantification of TGF-β (*n* = 4). **e**, qRT-PCR analysis of mRNA levels of *TGF-β1*, *Col-IV*, and *α-SMA* (*n* = 10). **f**, Renal MDA and SOD levels in STZ-diabetic mice (*n* = 7). **g**, Immunoblotting and quantification of c-Cas3, Bax, Nrf-2, and HO-1 (*n* = 6). **h**, Representative images and quantification of Col-IV staining (*n* = 5). **i**, Immunoblotting and quantification of TGF-β (*n* = 6). **j**, mRNA levels of *TGF-β1*, *Col-IV*, and *α-SMA* (*n* = 6). Data are presented as mean ± s.e.m. $^{*}p < 0.05$, $^{**}p < 0.01$, $^{***}p < 0.001$, $^{****}p < 0.0001$ as determined by unpaired two-tailed Student's *t*-test (**a**, **c**, **d**), ordinary two-way ANOVA followed by Sidak's multiple comparisons tests (**b**, **e**, **g**, **j**), or ordinary one-way ANOVA followed by Tukey's multiple comparisons tests (**f**, **h**, **i**). MDA, malondialdehyde; SOD, superoxide dismutase; c-Cas3, cleaved-Caspase-3; HO-1, heme oxygenase-1; Col-IV, collagen IV.

Supplementary Fig. 6

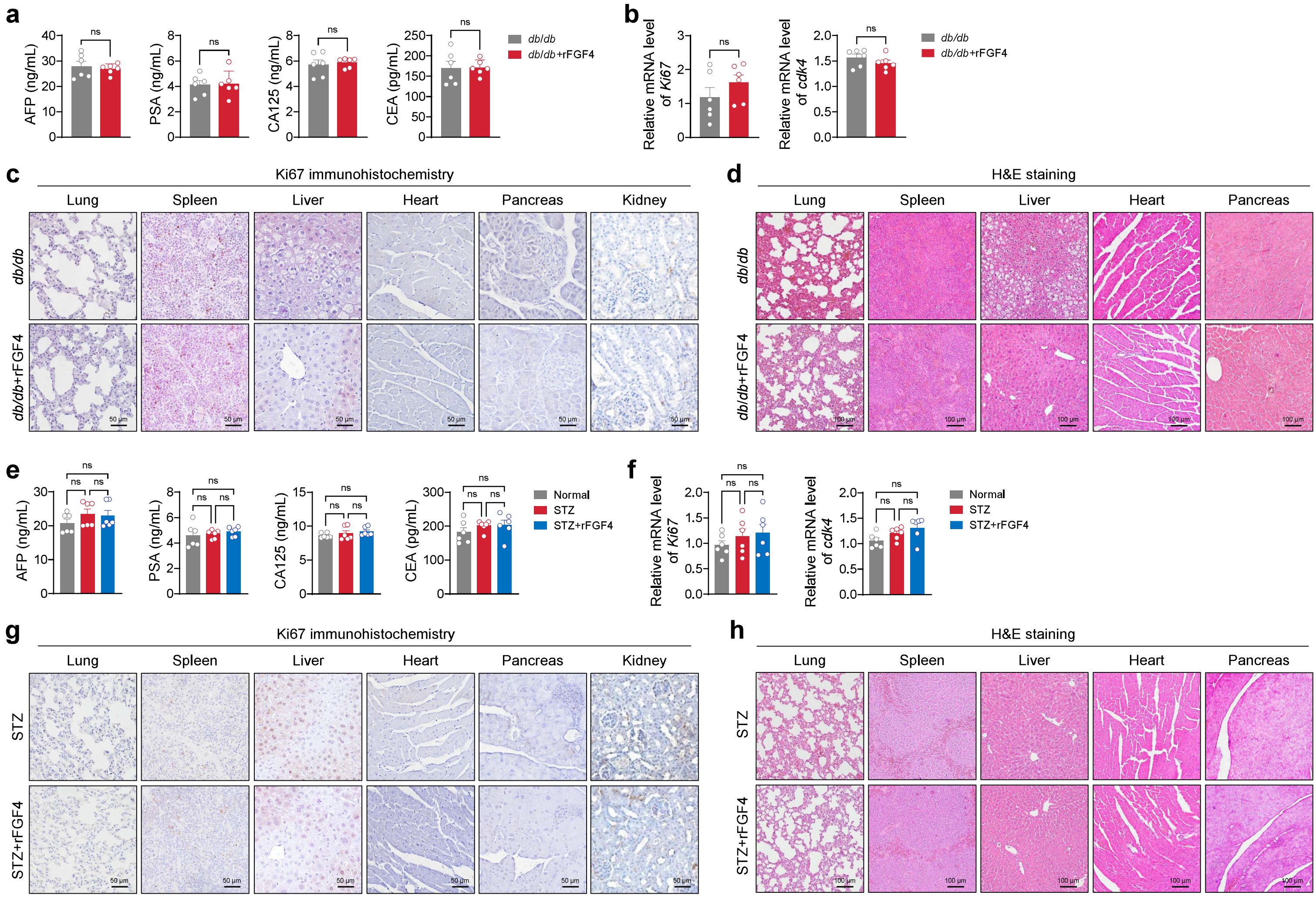

Supplementary Fig. 6. Safety evaluation of chronic rFGF4 administration in DKD models. a-h, Safety assessment of chronic rFGF4 administration in (**a-d**) *db/db* mice and (**e-h**) STZ-induced diabetic mice. **a**,**e**, Changes in serum tumor marker levels. **b**,**f**, Quantitative analysis of proliferation-related markers gene expression (*Ki67*, *Cdk4*) by RT-qPCR. **c**,**g**, Immunohistochemical staining of Ki67 in major organs. **d**,**h**, Representative H&E staining of major organs (heart, liver, spleen, pancreas, and lungs). *n* = 6 mice per group. Data are presented as mean ± s.e.m. ns, not significant. AFP, Alpha-Fetoprotein; PSA, Prostate-Specific Antigen; CA125, Carbohydrate Antigen 125; CEA, Carcinoembryonic Antigen.

Supplementary Fig. 7

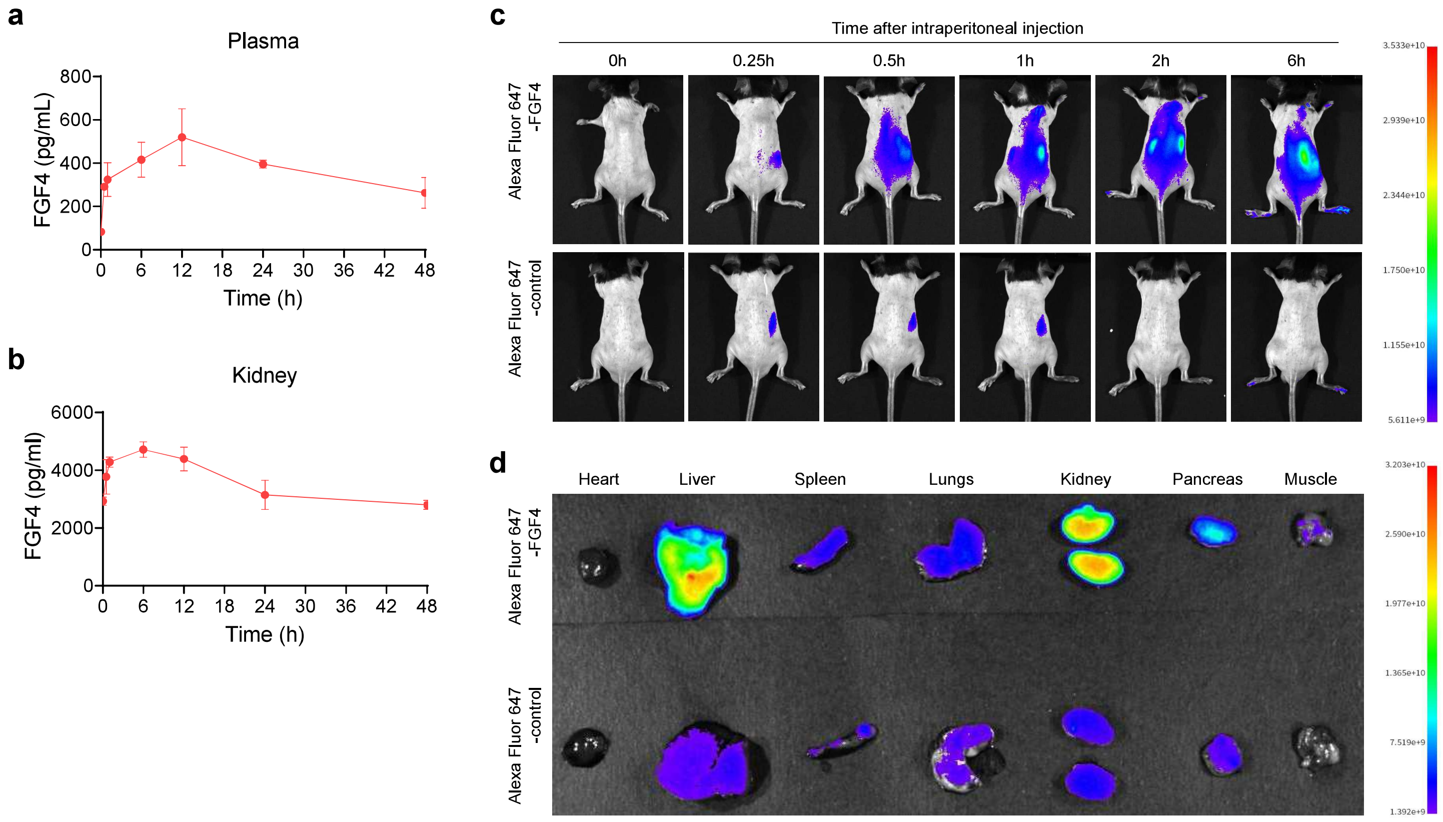

Supplementary Fig. 7. Pharmacokinetics and tissue distribution of fluorescently labeled FGF4. a, Serum concentration-time profile of rFGF4 measured by ELISA following intraperitoneal administration. **b**, Time-dependent accumulation of rFGF4 in renal tissues. **c**, *In vivo* fluorescence imaging of C57BL/6 mice at different time points after intraperitoneal injection of Alexa Fluor 647-FGF4, with unconjugated Alexa Fluor 647 control shown for comparison. **d**, *Ex vivo* fluorescence imaging of major organs harvested 6 hours post-administration, illustrating the biodistribution pattern of AF647-FGF4. Data are presented as mean ± s.e.m.

Supplementary Fig. 8

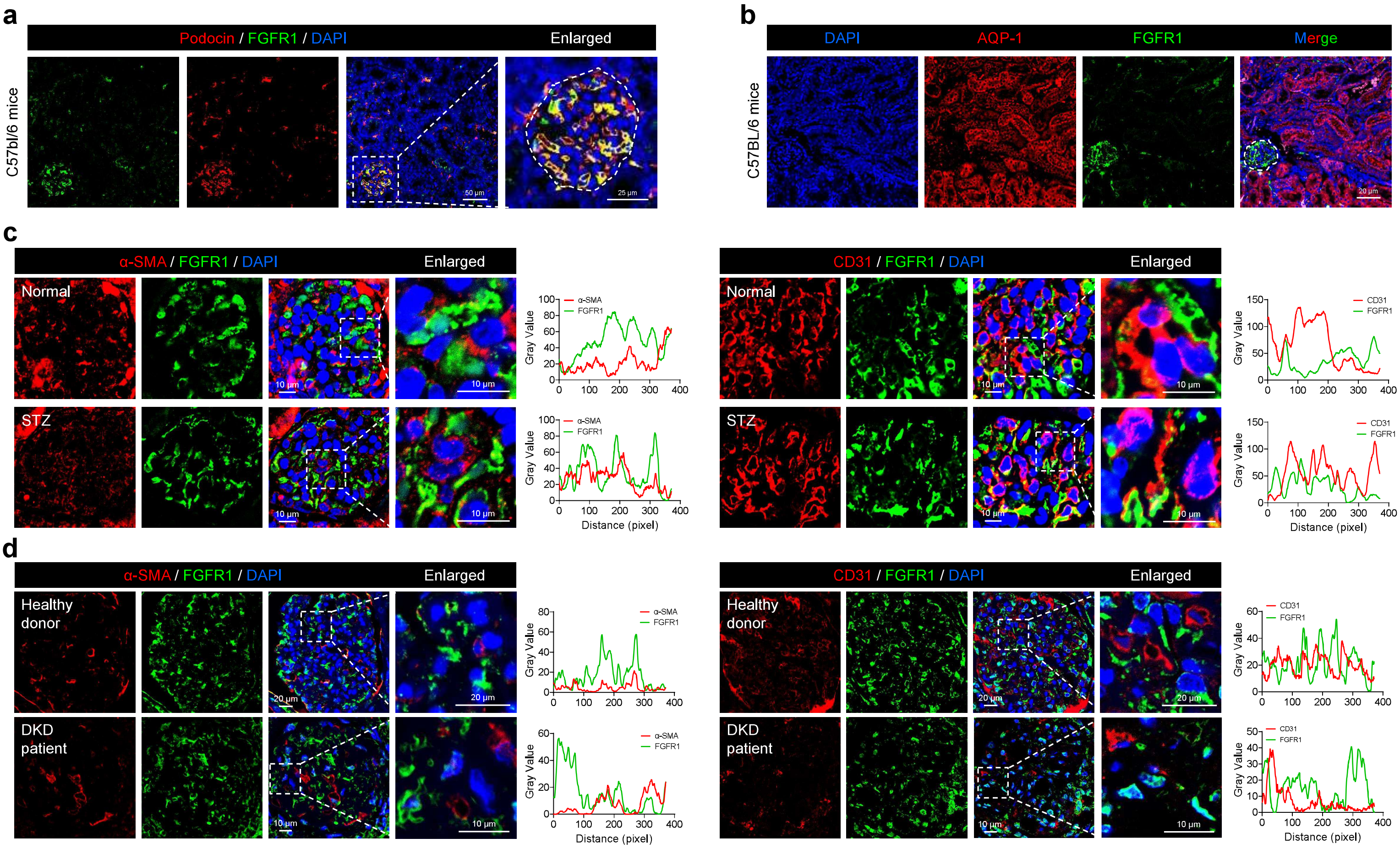

Supplementary Fig. 8. Expression profiling of FGFR1 in renal tissues under physiological and diabetic conditions. a,**b**, Immunofluorescence co-localization analysis of FGFR1 (green) with (**a**) podocin (podocyte marker, red) or (**b**) AQP-1 (tubular marker, red) in renal tissues. White dashed lines outline glomerular margins. **c**,**d**, Dual immunofluorescence staining showing FGFR1 (green) co-expression with α-SMA (red) or CD31 (red) in renal tissues from (**c**) STZ-induced diabetic mice and (**d**) human renal specimens. Nuclei were stained with DAPI (blue). AQP-1, aquaporin 1.

Supplementary Fig. 9

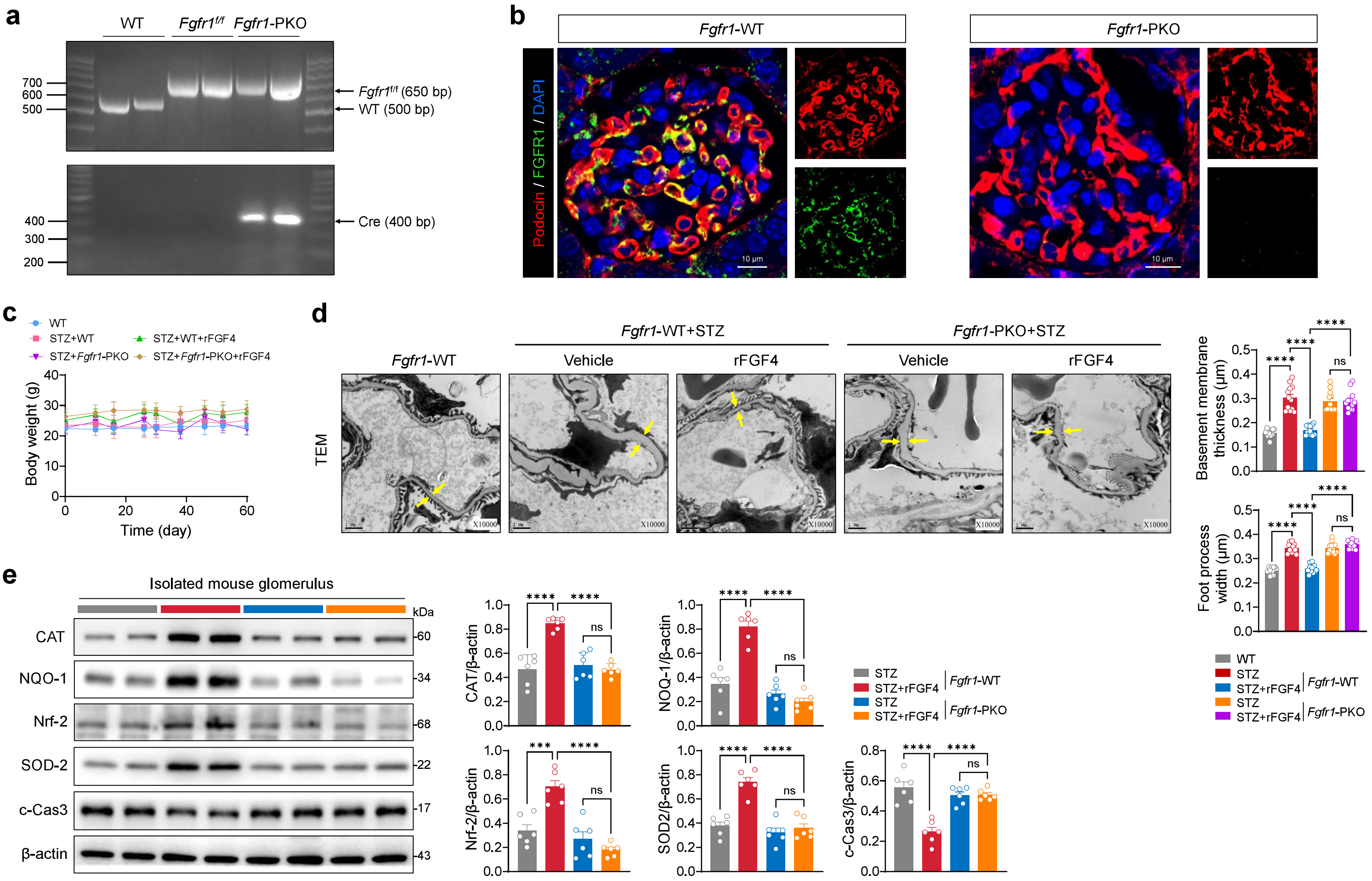

Supplementary Fig. 9. Role of FGFR1 in mediating therapeutic effects of FGF4 on renal podocytes. a, PCR genotyping confirms successful podocyte-specific *Fgfr1* knockout in conditional knockout mice. **b**, Immunofluorescence staining shows the near-complete absence of FGFR1 (green) in glomeruli knockout mice versus WT controls. **c**, Body weight changes across experimental groups. **d**, TEM images and quantification of renal. Yellow arrows refer to basement membranes. **e**, Immunoblotting and quantification of CAT, NQO-1, Nrf-2, SOD-2, and c-Cas3 in isolated mouse glomeruli from the designated groups (n = 6). β-actin was used as a loading control. Data are presented as mean ± s.e.m. $^{***}p < 0.001$, $^{****}p < 0.0001$ as determined by ordinary one-way ANOVA followed by Tukey's multiple comparisons tests (**d**, **e**); ns, not significant. bp, base pair; TEM, Transmission electron microscopy; c-Cas3, cleaved-Caspase3; CAT, Catalase.

Supplementary Fig. 10

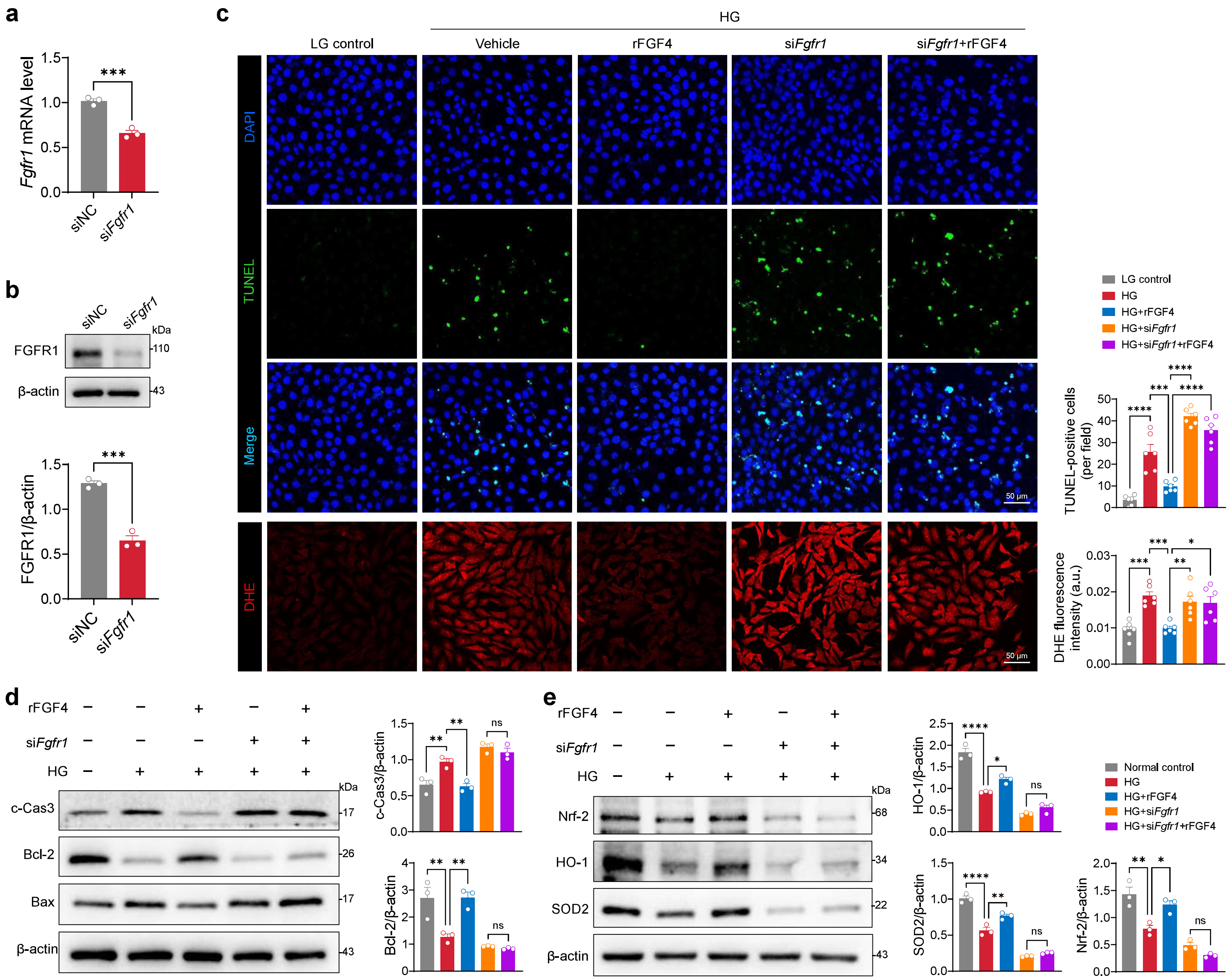

Supplementary Fig. 10. rFGF4-mediated enhancement of MPC-5 cell survival and stress resistance via FGFR1. a-e, MPC-5 cells were transfected with *Fgfr1* siRNA or control siRNA for 24 hours, followed by treatment with rFGF4 (100 ng/ml) or vehicle control and incubation with HG or LG for 24 hours. **a**,**b** Relative mRNA levels (**a**) and Western blot analysis (**b**) of FGFR1 in MPC-5 cells transfected with si*Fgfr1* or control siRNA (n = 3). **c**, Representative images and quantification of MPC-5 cells stained with TUNEL (green) and DHE (red) in the indicated groups (n = 6). Nuclei were stained with DAPI (blue). **d**,**e**, Western blot analysis and quantification of factors associated with apoptosis (**d**) and oxidative stress (**e**) in total MPC-5 cell lysates (n = 3). β-actin served as a loading controls. All data are presented as mean ± s.e.m. $^{*}p < 0.05$, $^{**}p < 0.01$, $^{***}p < 0.001$, and $^{****}p < 0.0001$ as determined by unpaired two-tailed Student's t-test (**a**, **b**) or ordinary one-way ANOVA followed by Tukey's multiple comparisons tests (**c**-**e**); ns, not significant. HG, high glucose; LG, low glucose.

Supplementary Fig. 11

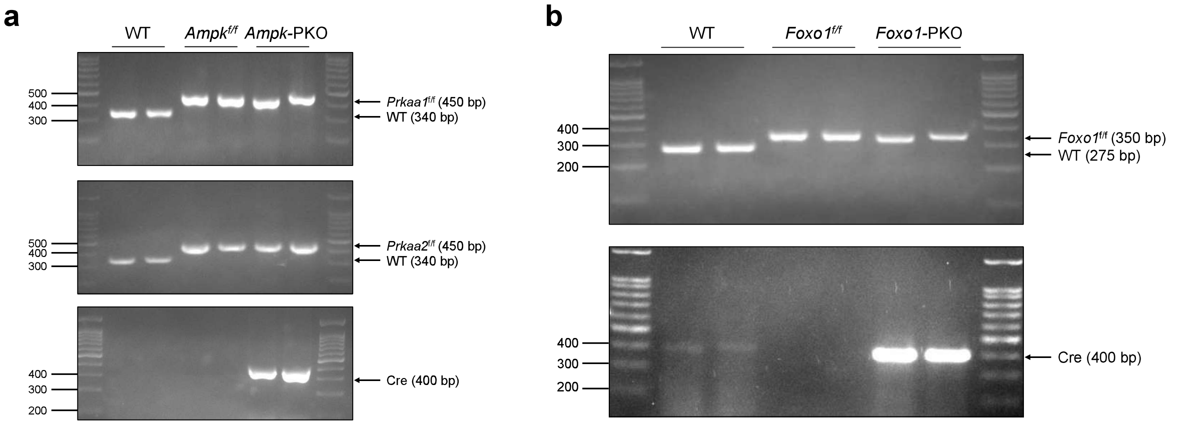

Supplementary Fig. 11. Genotyping of podocyte-specific *Ampk* and *Foxo1* knockout mice. **a**,**b**, PCR-based gene identification confirms successful knockout of (**a**) *Ampk* and (**b**) *Foxo1* in podocyte-specific conditional knockout mice. Representative gel electrophoresis images show wild-type (WT), floxed (fl/fl), and knockout (KO) genotypes for each target gene. bp, base pair.

Supplementary Fig. 12

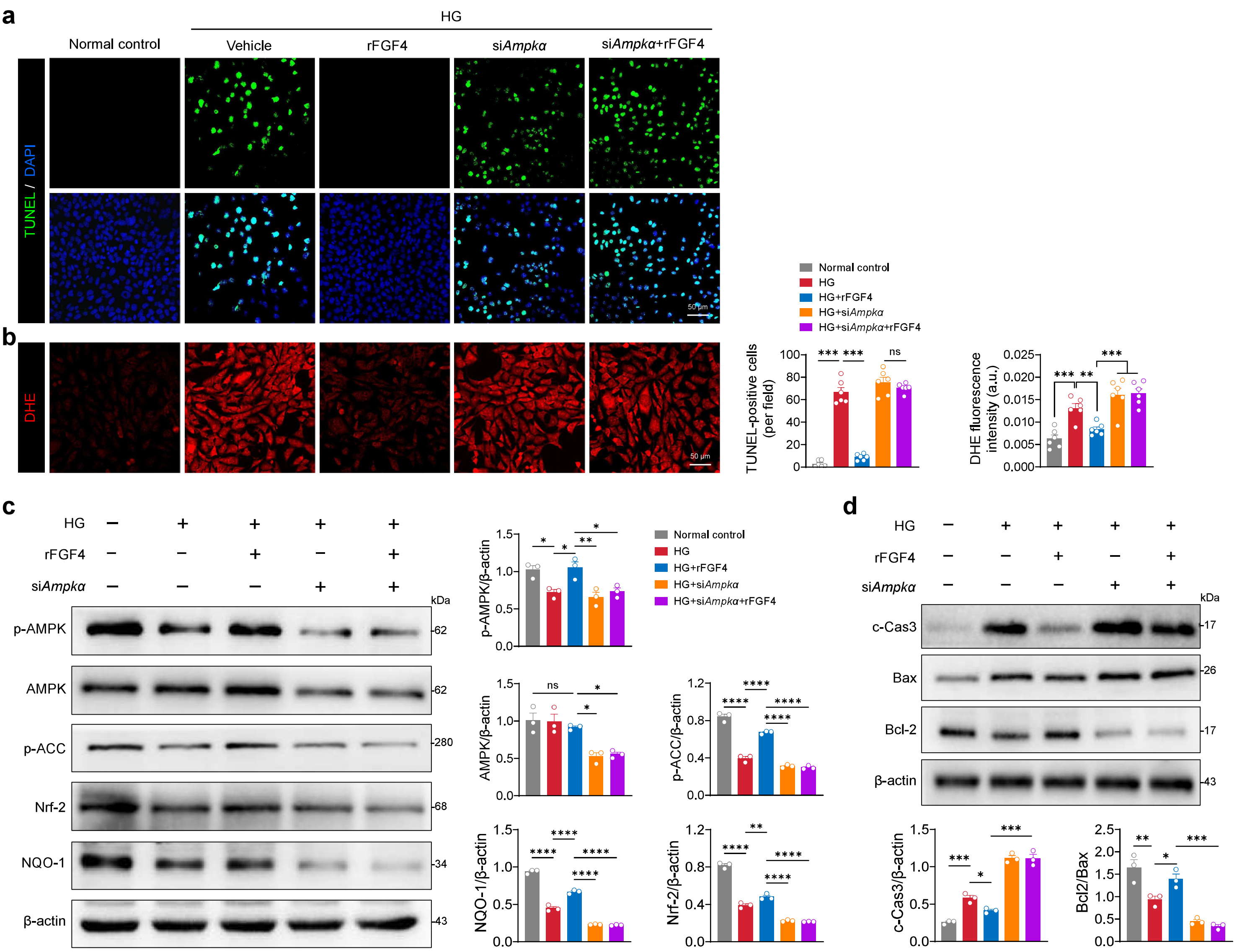

Supplementary Fig. 12. AMPK and FOXO1 knockout in MPC-5 cells abolishes the anti-apoptotic and antioxidant effects of rFGF4. a-d, MPC-5 cells were transfected with *Ampkα1/2* siRNA or *Foxo1* siRNA for 24 hours, followed by treatment with rFGF4 (100 ng/ml) or vehicle control. The cells were then incubated in high glucose or normal conditions for an additional 24 hours. **a**,**b**, Representative images and quantification of MPC-5 cells stained with TUNEL (**a**, green) and DHE (**b**, red) (n = 6). Nuclei were stained with DAPI (blue). **c**, Western blot analysis and quantification of the expression of phosphorylated (p)-AMPK, AMPK, p-ACC, Nrf-2, and NQO-1 expression in total MPC-5 cell lysates (n = 3). **d**, Western blot analysis and quantification of apoptosis-related markers (c-Cas3, Bax, and Bcl-2) in total MPC-5 cell lysates (n = 3). β-actin was used as a loading control. Data are presented as mean ± s.e.m. $^{*}p < 0.05$, $^{**}p < 0.01$, $^{***}p < 0.001$, $^{****}p < 0.0001$ as determined by ordinary one-way ANOVA followed by Tukey's multiple comparisons tests; ns, not significant. c-Cas3, cleved-Caspase3; CAT, Catalase.

Supplementary Fig. 13

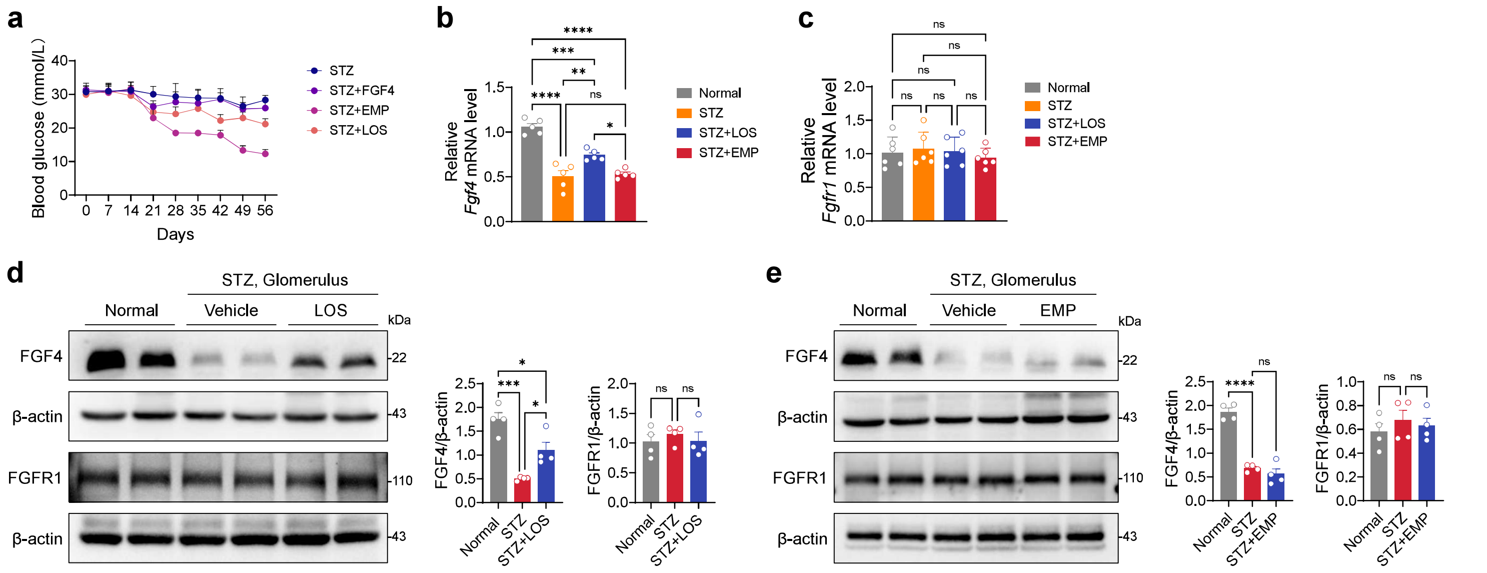

Supplementary Fig. 13. Therapeutic effects of RAS blockade and SGLT2 inhibitor on podocyte FGF4 and FGFR1 expression. a, Blood glucose levels across treatment groups. **b**,**c**, qRT-PCR analysis of (**b**) *Fgf4* (n = 5) and (**c**) *Fgfr1* (n = 6) mRNA levels in isolated glomeruli. **d**,**e**, Western blot analysis of FGF4 and FGFR1 protein expression in glomerular lysates from (**d**) LOS- and (**e**) EMP-treated mice (n = 4). β-actin was served as loading control. Data are presented as mean ± s.e.m. $^{*}p < 0.05$, $^{***}p < 0.001$, $^{****}p < 0.0001$ as determined by Ordinary one-way ANOYA followed by Tukey's multiple comparisons test. ns, not significant. LOS, Losartan potassium; EMP, Empagliflozin.

Supplementary Table 1. Detail information on human samples

Patients	Gender	Age	eGFR	Albuminuria	BUN	CRE	Sample	Remarks
**Diabetes without DKD**								
#1	Male	34-74	/	UACR: 19.04 mg/g Cr	/	/	Urine	Podocytes isolation
#2	Male		/	UACR: 15.68 mg/g Cr	/	/	Urine	
#3	Male		/	UACR: 17.64 mg/g Cr	/	/	Urine	
#4	Male		/	UACR: 17.36 mg/g Cr	/	/	Urine	
#5	Male		/	UACR: 13.44 mg/g Cr	/	/	Urine	
#6	Male		/	UACR: 14.28 mg/g Cr	/	/	Urine	
#7	Female		99.23	/	/	53.3	Urine	
#8	Male		120.28	UACR: 130.38 mg/g Cr	5.85	65.5	Urine	
#9	Male		91.42	UACR: 27.76 mg/g Cr	10.28	70.2	Urine	
#10	Male		94.83	UACR: 60.33 mg/g Cr		66.9	Urine	
#11	Male		93.21	UACR: 18.53 mg/g Cr	5.7	100.5	Urine	
#12	Male		97.36	UACR: 24.19 mg/g Cr	8.19	64.5	Urine	
#13	Male		114.84	/	5.48	58.7	Urine	
#14	Female		67.27	UACR: 242.17 mg/g Cr	14.24	103.6	Urine	
#15	Male		88.27	/	5.29	101.7	Urine	
**DKD patient**								
#1	Male	28-81	123	202	/	/	Urine	Podocytes isolation
#2	Male		211.4	203	/	/	Urine	
#3	Male		215.2	1102	/	/	Urine	
#4	Male		494.2	205	/	/	Urine	
#5	Male		238.1	1302	/	/	Urine	
#6	Male		49.38	1202	/	/	Urine	
#7	Male		/	121.35	/	/	Urine	
#8	Male		/	DKD III phase	/	/	Urine	
#9	Male		/	UACR: 633 mg/g Cr	/	/	Urine	
#10	Male		/	UACR: 346 mg/g Cr	/	/	Urine	
#11	Male		/	UACR: 10.49 mg/g Cr	/	/	Urine	
#12	Male		/	UACR: 184.78 mg/g Cr	/	/	Urine	
#13	Male		/	UACR: 238.1 mg/g Cr	/	/	Urine	
#14	Male		/	UACR: 248.82 mg/g Cr	/	/	Urine	
#15	Female		/	UACR: 494.2 mg/g Cr	/	/	Urine	
#16	Female		/	UACR: 215.2 mg/g Cr	/	/	Urine	
#17	Female		/	UACR: 871.5 mg/g Cr	/	/	Urine	
#18	Male		70.58	UACR: 115.14 mg/g Cr	6.6	60.1	Urine	
#19	Male		63.05	UACR: 291.29 mg/g Cr	7.6	101.3	Urine	
#20	Female		49.36	UACR: 200.32mg/g Cr	14	94.8	Urine	
#21	Male		57.02	UACR: 173.88mg/g Cr	11.1	121.5	Urine	
#22	Female		26.67	UACR: 316.16 mg/g Cr	12.3	202.8	Urine	
#23	Male		63.99	UACR: 491.04 mg/g Cr	8.6	112.4	Urine	
#24	Female		27.52	UACR: 547.91 mg/g Cr	15.1	149.3	Urine	
#25	Female		34.33	UACR: 408.22 mg/g Cr	11.1	164.6	Urine	
#26	Male		29.32	UACR: 637.15 mg/g Cr	12.2	197.6	Urine	
#27	Female		44.89	UACR: 705.17mg/g Cr	14.3	187.5	Urine	
#28	Male		24.25	UACR: 673.84mg/g Cr	6.7	176.7	Urine	
#29	Male		31.33	UACR: 851.09 mg/g Cr	17.1	156.9	Urine	
#30	Male		122	2650	7.52	122.8	Renal tissue	RNA-

#31	Female		13.1	1.84 (24 h albuminuria)	11.6	123.6	Renal tissue	Scope or IF analysis
#32	Female		/	9.44 (24 h albuminuria)	8.4	73.9	Renal tissue	
#33	Male		/	5.94 (24 h albuminuria)	13	109.8	Renal tissue	
#34	Male		/	1.8 (24 h albuminuria)	10.4	76.1	Renal tissue	
#35	Male		/	1.44 (24 h albuminuria)	6.8	87.1	Renal tissue	
#36	Female		/	2.79 (24 h albuminuria)	5.1	78.5	Renal tissue	
#37	Male		/	1020	8.1	94.4	Renal tissue	
#38	Female		/	UACR: 654.31 mg/g Cr	9.5	113.4	Renal tissue	
#39	Female		/	UACR: 481.45 mg/g Cr	7.8	90.5	Renal tissue	
**Healthy donor**								
#1	Female	44-71	/	Negative	4	55	Renal tissue	RNA-Scope analysis
#2	Male		/	Negative	6.4	88.5	Renal tissue	
#3	Male		/	Negative	4.9	63.7	Renal tissue	
#4	Male		/	Negative	7.6	76.4	Renal tissue	
#5	Male		/	Negative	6.4	72.7	Renal tissue	
#6	Female		/	Negative	5.4	59.4	Renal tissue	
#7	Female		/	Negative	4.9	51.2	Renal tissue	
#8	Male		/	Negative	5.7	67.4	Renal tissue	
#9	Male		/	Negative	7.2	61.2	Renal tissue	
#10	Male		/	Negative	6.6	58.7	Renal tissue	
#11	Female		/	Negative	6.4	81	Partial resection of right renal tumor	Glomeruli isolation
#12	Male		/	Negative	4.5	83		

DKD, Diabetic kidney disease; eGFR, estimated glomerular filtration rate; BUN, blood urea nitrogen; CRE, creatinine; UACR, urine albumin creatine ratio.

Supplementary Table 2. Detailed primers used in the qRT-PCR

Gene	Forward primer (5’-3’)	Reverse primer (5’-3’)
Fgf1	GGGGAGATCACAACCTTCGC	GTCCCTTGTCCCATCCACG
Fgf2	GCGACCCACACGTCAAACTA	TCCCTTGATAGACACAACTCCTC
Fgf3	TGCGCTACCAAGTACCACC	CACCGCAGTAATCTCCAGGAT
Fgf4	TGCCTTTCTTTACCGACGAGT	GCGTAGGATTCGTAGGCGTT
Fgf5	GTACGTGGCCCTGAACAAGA	CGGTGAAGGAAAGTTCCGGT
Fgf6	CAGGCTCTCGTCTTCTTAGGC	AATAGCCGCTTTCCCAATTCA
Fgf7	TGGGCACTATATCTCTAGCTTGC	GGGTGCGACAGAACAGTCT
Fgf8	GGAACCCAGCTGACACTCTC	TCTTCTGCCATGGCGTTGAT
Fgf9	ATGGCTCCCTTAGGTGAAGTT	TCCGCCTGAGAATCCCCTTT
Fgf10	TTTGGTGTCTTCGTTCCCTGT	TAGCTCCGCACATGCCTTC
Fgf16	GTGTTTTCCGGGAACAGTTTGA	GGTGAGCCGTCTTTATTCAGG
Fgf17	GCGGCAAATCCGTGAATACC	GGCCGTGTAGTTGTTCTCCA
Fgf18	GCCCTGATGTCTGCCAAGTA	CCCTTGGGGTAACGCTTCAT
Fgf15	GAAGACGATTGCCATCAAGGA	CGAATCAGCCCGTATATCTTGC
Fgf21	TTCAAATCCTGGGTGTCAAA	CAGCAGCAGTTCTCTGAAGC
Fgf22	GGAGATCCGTTCTGTCCGTG	TCCCGGAACCGACCCAT
Fgf23	ATGCTAGGGACCTGCCTTAGA	AGCCAAGCAATGGGGAAGTG
Bax	CCGGCGAATTGGAGATGAACT	CCAGCCCATGATGGTTCTGAT
Bcl-2	ATGCCTTTGTGGAACTATATGGC	GGTATGCACCCAGAGTGATGC
Col-4	CCTGGCACAAAAGGGACGA	ACGTGGCCGAGAATTTCACC
TGF-β	CCACCTGCAAGACCATCGAC	CTGGCGAGCCTTAGTTTGGAC
α-SMA	TCCTGACCCGAATCACCCAA	AGCTTCAGCAGTCTGTAGGTATT
Nrf-2	CTTTAGTCAGCGACAGAAGGAC	AGGCATCTTGTTTGGGAATGTG
NQO-1	AGGATGGGAGGTACTCGAATC	TGCTAGAGATGACTCGGAAGG
HO-1	AGGTACACATCCAAGCCGAGA	CATCACCAGCTTAAAGCCTTCT
Fgfr1	TAATACCACCGACAAGGAAATGG	TGATGGGAGAGTCCGATAGAGT
Fgfr2	GCCTCTCGAACAGTATTCTCCT	ACAGGGTTCATAAGGCATGGG
Fgfr3	TGGATCAGTGAGAATGTGGAGG	CCTATGAAATTGGTGGCTCGAC
Fgfr4	TTGGCCCTGTTGAGCATCTTT	GCCCTCTTTGTACCAGTGACG
Ki67	ATCATTGACCGCTCCTTTAGGT	GCTCGCCTTGATGGTTCCT

Cdk4	ATGGCTGCCACTCGATATGAA	TGCTCCTCCATTAGGAACTCTC
β-actin	GGCTGTATTCCCCTCCATCG	CCAGTTGGTAACAATGCCATGT
GAPDH	TGACCTCAACTACATGGTCTACA	CTTCCCATTCTCGGCCTTG
