# Supplementary material for: FGF4-FGFR1 signaling promotes podocyte survival and glomerular function to ameliorate diabetic kidney disease in male mice
Source: Nat Commun. 2025 Nov 25;16:10430. doi: 10.1038/s41467-025-65978-4 (PMC12647821; doi:10.1038/s41467-025-65978-4)
Supplement: Supplementary file 2 — Reporting Summary [file 41467_2025_65978_MOESM2_ESM.pdf]

nature portfolio

Corresponding author(s): Zhifeng Huang, Jian Sun, Jie Zhou, Chao Zheng

Last updated by author(s): Sep 13, 2025

Reporting Summary

Nature Portfolio wishes to improve the reproducibility of the work that we publish. This form provides structure for consistency and transparency in reporting. For further information on Nature Portfolio policies, see our Editorial Policies and the Editorial Policy Checklist.

Statistics

For all statistical analyses, confirm that the following items are present in the figure legend, table legend, main text, or Methods section.

n/a	Confirmed	
☐	☒	The exact sample size (*n*) for each experimental group/condition, given as a discrete number and unit of measurement
☐	☒	A statement on whether measurements were taken from distinct samples or whether the same sample was measured repeatedly
☐	☒	The statistical test(s) used AND whether they are one- or two-sided<br>*Only common tests should be described solely by name; describe more complex techniques in the Methods section.*
☒	☐	A description of all covariates tested
☒	☐	A description of any assumptions or corrections, such as tests of normality and adjustment for multiple comparisons
☐	☒	A full description of the statistical parameters including central tendency (e.g. means) or other basic estimates (e.g. regression coefficient) AND variation (e.g. standard deviation) or associated estimates of uncertainty (e.g. confidence intervals)
☐	☒	For null hypothesis testing, the test statistic (e.g. *F*, *t*, *r*) with confidence intervals, effect sizes, degrees of freedom and *P* value noted<br>*Give P values as exact values whenever suitable.*
☒	☐	For Bayesian analysis, information on the choice of priors and Markov chain Monte Carlo settings
☒	☐	For hierarchical and complex designs, identification of the appropriate level for tests and full reporting of outcomes
☒	☐	Estimates of effect sizes (e.g. Cohen's *d*, Pearson's *r*), indicating how they were calculated

Our web collection on statistics for biologists contains articles on many of the points above.

Software and code

Policy information about availability of computer code

Data collection	qRT-PCR data were acquired using Bio-Rad CFX Maestro 1.1 software (version 4.1.2433.1219); Western blot images were captured with Image Lab (version 5.1 build 8); Immunofluorescence images were acquired using NIS-Elements Viewer (version 5.21.00); Transmission electron microscopy (TEM) images were acquired using Hitachi version H-7500; ELISA results were acquired using SoftMax Pro software (Version7.0.1); RNA sequencing was conducted on an Illumina NovaSeq 6000 platform.
Data analysis	Image J (v1.52a) and GraphPad Prism 10 (version 10.1.2) were used.

For manuscripts utilizing custom algorithms or software that are central to the research but not yet described in published literature, software must be made available to editors and reviewers. We strongly encourage code deposition in a community repository (e.g. GitHub). See the Nature Portfolio guidelines for submitting code & software for further information.

Data

Policy information about availability of data

All manuscripts must include a data availability statement. This statement should provide the following information, where applicable:

- Accession codes, unique identifiers, or web links for publicly available datasets
- A description of any restrictions on data availability
- For clinical datasets or third party data, please ensure that the statement adheres to our policy

RNA-seq data have been deposited in the NCBI's Gene Expression Omnibus (GEO) under the accession number GSE287026 (https://www.ncbi.nlm.nih.gov/geo/query/acc.cgi?acc=GSE287026). The corresponding author provides further details upon reasonable request. Source data are provided with this paper.

Research involving human participants, their data, or biological material

Policy information about studies with human participants or human data. See also policy information about sex, gender (identity/presentation), and sexual orientation and race, ethnicity and racism.

Reporting on sex and gender	Male and female.
Reporting on race, ethnicity, or other socially relevant groupings	All human participants are Chinese. Race/ethnicity was self-reported and confirmed through clinical records. No additional socially relevant groupings were assessed.
Population characteristics	Renal biopsies from patients with chronic kidney disease had been performed as part of routine clinical diagnostic investigation. The samples of renal biopsies were obtained from The Second Affiliated Hospital and Yuying Children's Hospital of Wenzhou Medical University (n = 10). Control samples were obtained from the healthy kidney pole tissues of individuals who underwent tumor nephrectomies without diabetes or chronic renal disease (n = 12). Human urine samples were obtained from two groups of diabetic patients: non-DKD controls (defined as clinically diagnosed diabetic patients with persistently normal renal functional indicators; n = 15) and diagnosed DKD patients (n = 29). The urine samples were sourced from The Second Affiliated Hospital of Zhejiang University. Detail information on human samples are presented in Supplementary Table 1.
Recruitment	Participants were recruited from two hospitals in China: The Second Affiliated Hospital and Yuying Children's Hospital of Wenzhou Medical University and The Second Affiliated Hospital of Zhejiang University. All participants provided written informed consent. Recruitment bias was minimized by applying consistent diagnostic and exclusion criteria.
Ethics oversight	The study protocols were approved by the Institutional Ethics Committees of The Second Affiliated Hospital and Yuying Children's Hospital of Wenzhou Medical University (Approval No. 2025-K-93-02) and The Second Affiliated Hospital of Zhejiang University (Approval No. 2021-LSY-0479). All procedures involving human participants were conducted in compliance with the ethical principles outlined in the Declaration of Helsinki.

Note that full information on the approval of the study protocol must also be provided in the manuscript.

Field-specific reporting

Please select the one below that is the best fit for your research. If you are not sure, read the appropriate sections before making your selection.

☒ Life sciences ☐ Behavioural & social sciences ☐ Ecological, evolutionary & environmental sciences

For a reference copy of the document with all sections, see nature.com/documents/nr-reporting-summary-flat.pdf

Life sciences study design

All studies must disclose on these points even when the disclosure is negative.

Sample size	No statistical methods were used to predetermine sample size. Instead, sample sizes were estimated based on prior comparable studies and were sufficient to ensure reproducible differences between experimental groups. The number of independent replicates is specified in the figure legends, and a minimum of three biological replicates were used in all experiments.
Data exclusions	No data were excluded in this study.
Replication	All experiments were performed at least three times with similar results. All observations reported in the manuscript were reproducible.
Randomization	Sample allocation to each experimental group was randomized. The mice in the experiment were randomly selecting and divided into different groups.
Blinding	For mouse studies, blinding was not applicable as animals were grouped according to genotype and all samples were identified prior to analysis. For human subjects, blinding was not feasible due to the nature of the data; however, all analyses were conducted using pre-established protocols to minimize potential bias.

Reporting for specific materials, systems and methods

We require information from authors about some types of materials, experimental systems and methods used in many studies. Here, indicate whether each material, system or method listed is relevant to your study. If you are not sure if a list item applies to your research, read the appropriate section before selecting a response.

Materials & experimental systems

n/a	Involved in the study
☐	☒ Antibodies
☐	☒ Eukaryotic cell lines
☒	☐ Palaeontology and archaeology
☐	☒ Animals and other organisms
☒	☐ Clinical data
☒	☐ Dual use research of concern
☒	☐ Plants

Methods

n/a	Involved in the study
☒	☐ ChIP-seq
☒	☐ Flow cytometry
☒	☐ MRI-based neuroimaging

Antibodies

Antibodies used

Antibodies used for Western blot include:
FGF4 (1:1000, Cat. No. ab106355, Abcam), β-actin (1:1000, Cat. No. HC201-02, TransGen Biotech), Nephrin (1:1000, Cat. No. AF7951, Affinity), Podocin (1:1000, Cat. No. sc518088, Santa Cruz), TGF-β1 (1:1000, Cat. No. ab215715, Abcam), cleaved caspase3 (1:1000, Cat. No. TA7022S, Abmart), Bax (1:1000, Cat. No. sc493, Santa Cruz), Nrf-2 (1:5000, Cat. No. 16396, Proteintech), HO-1 (1:2000, Cat. No. ab13243, Abcam), FGFR1 (1:1000, Cat. No. 9740S, Cell Signaling), phospho-AMPKα (1:1000, Cat. No. 2535, Cell Signaling), AMPKα (1:1000, Cat. No. ab32047, Abcam), FOXO1 (pS256) (1:1000, Cat. No. PA5293, Abmart), FoxO1a (1:1000, Cat. No. T55376, Abmart), catalase (1:1000, Cat. No. A11777, ABclonal), SOD2 (1:1000, Cat. No. PK08370, Abmart), NQO-1 (1:1000, Cat. No. 67240, Proteintech), Bcl-2 (1:1000, Cat. No. T40056, Abmart), phospho-ACC (1:1000, Cat. No. 11818, Cell Signaling), and Histone H3 (1B1B2) (1:1000, Cat. No. 14269, Cell Signaling).
Antibodies used for Immunohistochemistry include:
Wilms Tumor (1:50, Cat. No. ab89901, Abcam), Nephrin (1:1000, Cat. No. ab216341, Abcam), NPHS2 (1:300, Cat. No. ab181143, Abcam), collagen IV (1:400, Cat. No. ab6586, Abcam), and Ki67 (1:2000, Cat. No. 28074-1-AP, Proteintech).
Antibodies used for Immunofluorescence staining include:
FGF4 (1:400, Cat. No. 106355, Abcam), Podocin (1:100, Cat. No. sc518088, Santa Cruz), FoxO1a (1:200, Cat. No. T55376, Abmart), CD31 (1:200, Cat. No. 66065, Proteintech), β-Tubulin (C66) (1:1000, Cat. No. M20005, Abmart), FGFR1 (1:300, Cat. No. ab824, Abcam), Alpha-smooth muscle actin (1:300, Cat. No. 124964, Abcam), Wilms Tumor (1:50, Cat. No. ab89901, Abcam), AQP-1 (1:250, Cat. No. AF5231, affinity) and Nrf-2 (1:200, Cat. No. ab62352, Abcam), FGFR1 (1:1000, Cat. No. 60325, Proteintech), and NPHS2 (1:1000, Cat. No. 20384, Proteintech).

Validation

FGF4 antibody: https://www.abcam.cn/products/primary-antibodiefgf4-antibody-ab106355.html
β-actin antibody: https://www.clinisciences.com/en/other-products-186/proteinfind-anti-actin-mouse-monoclonal-291000343.html
Nephrin antibody: https://www.affbiotech.cn/goods-14597-AF7951-Nephrin_Antibody.html
Podocin antibody: https://www.scbt.com/p/podocin-antibody-g-5
TGF-β1 antibody: https://www.abcam.cn/products/primary-antibodies/tgf-beta-1-antibody-epr21143-ab215715.html
cleaved caspase3 antibody: https://www.ab-mart.com.cn/product.aspx?id=3&f=cn&keys=TA7022S
Bax antibody: https://www.scbt.com/p/bax-antibody-n-20
Nrf-2 antibody: https://www.ptgcn.com/products/NFE2L2,NRF2-Antibody-16396-1-AP.htmHO-1 antibody: https://www.abcam.cn/products/primary-antibodies/heme-oxygenase-1-antibody-ab13243.html
FGFR1 antibody: https://www.cellsignal.cn/products/primary-antibodies/fgf-receptor-1-d8e4-xp-rabbit-mab/9740
phospho-AMPKα antibody: https://www.cellsignal.cn/products/primary-antibodies/phospho-ampka-thr172-40h9-rabbit-mab/2535
AMPKα antibody: https://www.abcam.cn/products/primary-antibodies/ampk-alpha-1-antibody-y365-ab32047.html
FOXO1 (pS256) antibody: https://www.ab-mart.com.cn/product.aspx?id=3&f=cn&keys=PA5293
FoxO1a antibody: https://www.ab-mart.com.cn/product.aspx?id=3&f=cn&keys=T55376
Catalase antibody: https://abclonal.com.cn/catalog/A11777
SOD2 antibody: https://www.ab-mart.com.cn/product.aspx?id=3&f=cn&keys=PK08370
NQO-1 antibody: https://www.ptgcn.com/products/NQO1-Antibody-67240-1-Ig.htm
Bcl-2 antibody: https://www.ab-mart.com.cn/Product.aspx?cat=&keys=T40056
phospho-ACC antibody: https://www.cellsignal.cn/products/primary-antibodies/phospho-acetyl-coa-carboxylase-ser79-d7d11-rabbit-mab/11818
Histone H3 (1B1B2) antibody: https://www.cellsignal.cn/products/primary-antibodies/histone-h3-1b1b2-mouse-mab/14269
Wilms Tumor antibody: https://www.abcam.cn/products/primary-antibodies/wilms-tumor-protein-antibody-can-r9ihc-56-2-ab89901.html
Nephrin antibody: https://www.abcam.cn/products/primary-antibodies/nephrin-antibody-epr20993-ab216341.html
NPHS2 antibody: https://www.abcam.cn/products/primary-antibodies/nphs2-antibody-epr13820-ab181143.html
collagen IV antibody: https://www.abcam.cn/products/primary-antibodies/collagen-iv-antibody-ab6586.html
Ki67 antibody: https://www.ptgcn.com/products/ki67-Antibody-28074-1-AP.htm
FGFR1 antibody: https://www.abcam.cn/products/primary-antibodies/fgfr1-antibody-m5g10-ab824.html
Alpha-smooth muscle actin antibody: https://www.abcam.cn/products?keywords=124964

Eukaryotic cell lines

Policy information about cell lines and Sex and Gender in Research

Cell line source(s)	The MPC-5 cell line was obtained from Procell (Wuhan, China; Cat. No. CL-0855); and primary podocytes were isolated from human clinical urine samples.
Authentication	The cell lines used in this study were authenticated by the supplier using short tandem repeat (STR) profiling.
Mycoplasma contamination	All cell lines were tested for mycoplasma contamination and confirmed to be negative.
Commonly misidentified lines (See ICLAC register)	No commonly misidentified cell lines were used in this study.

Animals and other research organisms

Policy information about studies involving animals; ARRIVE guidelines recommended for reporting animal research, and Sex and Gender in Research

Laboratory animals	Male C57BL/6J, db/db (C57BLKS/J-leprdb/leprdb), and their nondiabetic db/dm littermates (8–10 weeks old) were purchased from the Model Animal Research Center of Nanjing University (Nanjing, China). Fgf4-floxed mice (Fgf4flox/flox, Cat. No. T009264) were obtained from GemPharmatech Co., Ltd. (Jiangsu, China), while Fgfr1-flox mice (Fgfr1flox/flox) and podocin (Pod)-Cre mice were acquired from Cyagen Biosciences Inc. (Suzhou, China). Foxo1-Flox mice (Foxo1flox/flox, Cat. No. NM-CKO-200177) were purchased from Shanghai Model Organisms Center (Shanghai, China). Ampkα1/α2-floxed (Ampkα1/α2flox/flox) mice were generously provided by Dr. Sheng-Cai Lin (Xiamen University, Fujian, China). Fgfr1flox/flox, Ampkα1/α2flox/flox, and Foxo1flox/flox mice were separately bred with Pod-Cre transgenic mice, resulting in the development of podocin-specific Fgfr1 knockout (Fgfr1flox/flox; Pod-Cre+, Fgfr1-PKO), Ampkα1/α2 double knockout (Ampkflox/flox; Pod-Cre+, Ampk-PKO), and Foxo1 knockout (Foxo1flox/flox; Pod-Cre+, Foxo1-PKO) mice. All mice were acclimated to the laboratory conditions and were housed in a controlled environment (22 ± 2 °C, 50–60% humidity, and 12-h light/dark cycle with lights on at 7 AM), with ad libitum access to food and water.
Wild animals	The study did not involve wild animals.
Reporting on sex	The study included only male mice. Sex was determined based on physical examination for the mice.
Field-collected samples	The study did not involve samples collected from the field.
Ethics oversight	All animal protocols were approved by the Institutional Animal Care and Use Committee at Wenzhou Medical University.

Note that full information on the approval of the study protocol must also be provided in the manuscript.

Plants

Seed stocks	No plants were used in this study.
Novel plant genotypes	No plants were used in this study.
Authentication	No plants were used in this study.
